# Kinetic and Sequence-Structure-Function Analysis of LinB Enzyme Variants with β- and δ-Hexachlorocyclohexane

**DOI:** 10.1371/journal.pone.0103632

**Published:** 2014-07-30

**Authors:** Rinku Pandey, Del Lucent, Kirti Kumari, Pooja Sharma, Rup Lal, John G. Oakeshott, Gunjan Pandey

**Affiliations:** 1 CSIRO Ecosystem Sciences, Acton, ACT, Australia; 2 CSIRO Center for Materials Science and Engineering, Parkville VIC, Australia; 3 Division of Engineering and Physics, Wilkes University, Wilkes-Barre, Pennsylvania, United States of America; 4 Department of Zoology, University of Delhi, Delhi, India; University of Toulouse - Laboratoire d’Ingénierie des Systèmes Biologiques et des Procédés, France

## Abstract

Organochlorine insecticide hexachlorocyclohexane (HCH) has recently been classified as a ‘Persistent Organic pollutant’ by the Stockholm Convention. The LinB haloalkane dehalogenase is a key upstream enzyme in the recently evolved Lin pathway for the catabolism of HCH in bacteria. Here we report a sequence-structure-function analysis of ten naturally occurring and thirteen synthetic mutants of LinB. One of the synthetic mutants was found to have ∼80 fold more activity for β- and δ-hexachlorocyclohexane. Based on detailed biophysical calculations, molecular dynamics and ensemble docking calculations, we propose that the latter variant is more active because of alterations to the shape of its active site and increased conformational plasticity.

## Introduction

Isomers of hexachlorocyclohexane (HCH) have been widely used as insecticides in various agricultural systems around the world for most of the last 70 years [1], [2]. Despite mounting concerns about their human and eco-toxicity, they still find heavy use in some developing countries in particular [2], [3]. Only the γ isomer is insecticidal but either a mixture of the four major isomers (α, β, γ and δ), known as technical HCH, or purified γ-HCH, known as lindane, have been used commercially. The purification of lindane from technical HCH has led to massive dumps (>50,000 tonnes) of the other isomers in several countries and the stability of all isomers (but particularly of β-HCH) has led to widespread contamination of the environment, originating both from the dump sites and broad scale agricultural uses [1]–[4].

Over 60 bacterial strains which can degrade HCH, about half of them Sphingomonads, have now been reported, and all those characterized biochemically and genetically have proven to use the well established Lin pathway (encoded by various *lin* genes) to degrade HCH [2], [5], [6]. There are two major forms of the pathway which differ in their initial reactions but subsequently converge. One form operates on α-, γ- and δ-HCH and is initiated by two rounds of dehydrochlorination, followed by two rounds of hydrolytic dechlorination. The other form of the pathway operates on β- and δ-HCH and is initiated by two rounds of hydrolytic dechlorination, albeit some of the subsequent steps remain to be elucidated. The *linA*/LinA gene/enzyme system catalyzes dehydrochlorinations in both forms of the pathway via an E2 reaction mechanism, and the *linB*/LinB gene/enzyme system catalyzes the hydrolytic dechlorinations via S_N_2 displacement reactions [2], [5], [6].

The LinB enzyme is a monomeric 32 kDa protein from the α/β hydrolase fold superfamily [7]. Its structure has been solved [8] and its catalytic mechanism is reasonably well understood, with an Asp-His-Glu catalytic triad mediating nucleophilic attack on the substrate and then on an acyl-enzyme intermediate, and an oxyanion hole stabilizing the intermediate [9]–[12]. The best characterized LinB, LinB_UT26A_ from *Sphingobium japonicum* strain UT26, has a broad substrate specificity, mainly due to a large active site volume, which includes monochloroalkanes (C3–C10), dichloroalkanes, bromoalkanes and chlorinated aliphatic alcohols [7], [10]. Notably, this variant yields a significantly lower specificity constant for β-HCH (0.02 mM^−1^ s^−1^) as compared to another relatively well characterized LinB, namely, LinB_B90A_ (identical to LinB_MI1205_, and LinB_BHC-A_ and LinB_pLB1_) from *Sphingobium indicum* strain B90A (0.20 mM^−1^ s^−1^) [13]. Nonetheless the activity of LinB_B90A_ for β-HCH is much lower than that of LinB_UT26A_ for some of the other halogenated aliphatic compounds mentioned above (see [7], [13] for a comprehensive list of kinetic constants).

A total of ten naturally occurring LinB variants have now been described which differ by as many as 16 (5.4%) of their amino acid residues [2]. At least some of these variants differ qualitatively in their substrate specificities; in addition to the β- and δ-HCH difference above, LinB_B90A_ will hydrolytically dechlorinate the metabolite tetrachlorocyclohenol (TCDL), whereas LinB_UT26_ does not [14]. A molecular dynamics simulation study suggests that this is mainly due to a difference in the flexibility of the entrance of the substrate access tunnel mediated by six out of the seven amino acid differences between the two enzyme variants [13].

Given the low but quantitatively different activities of the best characterized LinB_UT26_ and LinB_B90A_ variants for β-and δ-HCH, and their quantitative differences in respect to TCDL, our aim in the work described herein has been to quantitatively assess the variation in the β- and δ-HCH activities of all ten known naturally occurring LinB variants, plus another 13 synthetic variants derived on the basis of these data and the known structure of the LinB_UT26_ protein. We find one synthetic variant with nearly 80-fold higher activity than LinB_B90A_ for β-HCH and we suggest an explanation for its performance on the basis of increased mobility of its cap domain and increased affinity for the substrate.

## Materials and Methods

### Gene synthesis, expression vectors and chaperones

Codon optimized *linB* genes for expression in *E. coli* were synthesized by Geneart AG, Regensburg Germany (Table S1). These genes were PCR amplified with respective attB1, attB2 and attB2-R2 primers (Table S2) and the amplicons were then cloned into pDONR201 and transferred to pDEST17, following the manufacturer’s instructions (Invitrogen, CA). The host *E. coli* BL21-AI (Invitrogen) cells for some clones co-expressed chaperones from the plasmid pGro7 (Takara, Japan).

### Gene expression, enzyme purification and enzyme assays

Gene expression, enzyme purification and enzyme assays were performed as described earlier [15]. Briefly, the expression clones were grown in LB at 28°C until the OD_600_ has reached 0.5. At this point L-(+) arabinose was added at a final concentration of 2 g/L. Cultures were grown overnight, cells were harvested by centrifugation and cell free extract was prepared in 10 mM imidazole buffer containing 1X Bugbuster solution (Novagen, Darmstadt). The cell free extract was centrifuged at 16,000 g for 20 min at 4°C and the supernatant was subject to the Ni^2+^-affinity chromatography to purify 6xHis-tagged enzyme using standard procedures. The purified enzymes were quantified using Nanodrop (Thermo Scientific, DE) and stored in storage buffer (pH 7.5) containing 10% glycerol and 1 mM 2-mercaptoethanol at 4°C. Enzyme assays were performed within 3 days of purification in a 500 µl reaction mixture (final volume) containing 1.7 µM of HCH in Tris glycine buffer (25 mM Tris, 193 mM Glycine, pH 8.3) at 22°C. The reaction was initiated by adding enzyme and terminated by adding 0.3% formic acid (final concentration). HCH depletion in the reaction was monitored by an electron capture detector coupled to a gas chromatograph as described earlier [15]. The enzyme assays were conducted in triplicate.

### Computational analysis

Atomistic models of LinB_B90A_ as well as our most active mutant, LinB_G2.2_, were constructed by performing *in silico* mutagenesis on the LinB_UT26_ structure (pdb 2BFN, [12]) with the Rosetta software suite. The appropriate residues were mutated and the surrounding non-catalytic residues were allowed to repack in accordance with the Rosetta fixed backbone protein design protocol [16]. During these calculations, the catalytic residues were kept in their crystallographic conformations while 500 cycles of repacking and optimization were performed and the lowest energy structure was retained. To construct models with β-HCH bound, the Rosetta enzyme design protocol was used to dock the transition state structure into the active site [17]. Restraints were added to keep β-HCH as close to the transition state as possible (the transition state was obtained from Brittain *et al.* based on their previous characterization of this reaction using electronic structure calculations [18]). Again, 500 cycles of repacking and optimization were performed and the lowest energy result was used. The reactant structure of β-HCH was fitted onto the transition state complex to yield a final conformation that was geometrically well posed for catalysis and still well described by a classical molecular mechanics energy function (Figure S1).

In order to assess the approximate affinity of LinB_B90A_ and LinB_G2.2_ for β-HCH, the Rosetta Ligand Dock program [19] was used to dock β-HCH into the apo structures of each enzyme. For each enzyme, 500 docking trajectories were computed. The results were first ranked by their total score (which approximates the overall stability of the model). The top 5% of these docked complexes were then ranked by their binding energy for β-HCH. The average binding energy was computed from the 10 best models.

Atomistic molecular dynamics simulations were performed on the apo and β-HCH-bound states of LinB_B90A_ and LinB_G2.2_. These simulations were performed using the GROMACS 4.5.3 simulation package [20] (default parameters are used unless otherwise specified) with the AMBER99sb force field [21], the generalized Amber force field for β-HCH parameters [22], and the TIP3P explicit water model [23]. Temperature was kept at 298°K using the velocity-rescaling thermostat [24]. A 2-femtosecond timestep was used for all dynamics simulations with bond lengths constrained using the SHAKE algorithm [25]. Electrostatic interactions were calculated using the particle-mesh-Ewald method [26] with a cutoff of 15 Å. After minimization, simulations were equilibrated by running for 0.5 ns at constant volume and temperature with the positions of all non-solvent atoms harmonically restrained. Following this equilibration, the restraints were removed and a 30 ns production run was performed. Results were analysed using the VMD software package [27] and 3D structures were rendered using PyMOL (The PyMOL Molecular Graphics System, Version 1.3, Schrödinger, LLC).

## Results and Discussion

Eight of the ten natural LinB variants showed clearly measurable activities with both β- and δ-HCH (Table 1). As expected from previous reports, LinB_UT26_ yielded lower activities with either isomer [7], [13], [14], as did the closely related LinB_SP+_. The other eight variants all showed 10–20 fold greater activities with β- than δ-HCH.

**Table 1:** Activities towards β- and δ-HCH of the ten known naturally occurring LinB variants (organised according to the three sequence-based groups recognised by Lal et al [2], the thirteen synthetic mutants made herein, and the eight variants analysed by Ito et al [14].

LinB variants			Haplotypes																Turnover number (min^−1^)		
			2	6	7	81	83	112	134	135	138	147	150	222	223	224	247	253		β-HCH	δ-HCH
***Group 3***	W1.10	LinB_SS04-1_	.	.	.	A	.	.	L	A	I	.	.	.	.	.	S	.		74±7	4±1
	W1.9	LinB_SS04-2_	.	.	.	A	.	A	L	A	I	.	.	.	.	.	S	.		177±9	3±1
	W1.8	LinB_SS04-5_	.	.	.	.	P	A	L	A	I	.	.	.	.	.	S	M		214±13	6±1
	W1.7	LinB_Sp+_	.	.	.	.	.	A	L	A	I	.	.	.	.	.	S	M		0*	0*
	W1.6	LinB_UT26_	.	.	.	A	.	A	I	A	I	.	.	.	.	.	A	M		0*	0*
***Group 2***	W1.5	LinB_NM05_	I	N	A	A	.	.	.	.	.	Y	M	V	H	.	A	M		124±13	6±1
***Group 1***	W1.4	LinB_SS04-3_	.	.	.	A	.	.	.	.	.	.	.	.	.	.	S	.		170±15	8±1
	W1.3	LinB_ITRC-5-A_	.	.	.	A	.	.	.	.	.	.	.	.	.	.	.	.		154±31	8±1
	W1.2	LinB_ITRC-5-B_	.	.	.	A	.	.	.	.	.	.	.	.	.	V	S	.		65±9	5±0
	W1.1	LinB_B90A_	S	K	P	T	A	V	V	T	L	D	L	A	I	A	H	I		90±1	4±1
**Synthetic variants**	G1.1	A83P	.	.	.	.	P	.	.	.	.	.	.	.	.	.	.	.		182±17	6±1
	G1.2	V134L	.	.	.	.	.	.	L	.	.	.	.	.	.	.	.	.		138±10	9±2
	G1.3	T135L	.	.	.	.	.	.	.	L	.	.	.	.	.	.	.	.		194±3	10±2
	G1.4	L138I	.	.	.	.	.	.	.	.	I	.	.	.	.	.	.	.		9±1	11±2
	G1.5	H247S	.	.	.	.	.	.	.	.	.	.	.	.	.	.	S	.		1±1	0*
	G1.6	I253M	.	.	.	.	.	.	.	.	.	.	.	.	.	.	.	M		20±1	0*
	G1.7	A83P	.	.	.	A	P	.	.	.	.	.	.	.	.	.	S	.		17±2	0*
	G1.8	A247H	I	N	A	A	.	.	.	.	.	Y	M	V	H	.	H	M	C	24±4	5±1
	G2.1	T81A/A83P	.	.	.	A	P	.	.	.	.	.	.	.	.	.	.	.		44±6	4±1
	G2.2	V134L/T135L	.	.	.	.	.	.	L	L	.	.	.	.	.	.	.	.	C	6920±272	241±6
	G2.3	V134L/T135L/T81A	.	.	.	A	.	.	L	L	.	.	.	.	.	.	.	.	C	422±12	31±6
	G2.4	L138I/H247S/I253M	.	.	.	.	.	.	.	.	I	.	.	.	.	.	S	M	C	10±2	3±1
	G2.5	A81T/A83P	.	.	.	.	P	.	.	.	.	.	.	.	.	.	S	.	C	4±1	2±1
																			**Activity (mM** ^−**1**^ ** min^1^)**		
		LinB_UT26_	.	.	.	A	.	A	L	A	I	.	.	.	.	.	A	M		1.626	ND
**Mutations** **from Ito** **et al.** [13]		I134V	.	.	.	A	.	A	V	A	I	.	.	.	.	.	A	M		5.814	ND
		A247H	.	.	.	A	.	A	L	A	I	.	.	.	.	.	H	M		0.462	1.626
		I134V/A247H	.	.	.	A	.	A	V	A	I	.	.	.	.	.	H	M		1.458	ND
		LinB_MI1205/B90A_	.	.	.	.	.	.	.	.	.	.	.	.	.	.	.	.		12.30	ND
		V134I	.	.	.	.	.	.	I	.	.	.	.	.	.	.	.	.		7.44	ND
		H247A	.	.	.	.	.	.	.	.	.	.	.	.	.	.	A	.		12.6	1.23
		V134I/H247A	.	.	.	.	.	.	I	.	.	.	.	.	.	.	A	.		6.24	ND

The LinB_B90A_ is used as a reference sequence. All generation one (G1) and generation two (G2) mutants were derived from LinB_B90A_, except G1.7 and G1.8 which were derived from LinB_SS04-3_ and LinB_NM05_, respectively. Activities determined herein are given as turnover numbers (±standard deviation) (min^−1^) and those from Ito et al [14] as specific activities (mM^−1^ min^−1^). C indicates variants whose expression required the use of chaperones (see Material and Methods). ND = not determined. 0* = <1 min^−1^.

There was about a three-fold range of activities for each isomer across the latter eight variants. However, there was no obvious difference in activities across the three sequence-based groups of variants recognized by Lal et al [2] (again excluding LinB_UT26_ and LinB_SP+_). Instead, there was considerable variation within their two major groups (1 and 3 in Table 1), including some large differences between otherwise very closely related variants. The sequence differences between these variants frequently involved residues (eg 112, 134, 135, 138 and 253) constituting the enzyme’s active site.

Based on the above results, six generation one synthetic variants (denoted G1.1–G1.6) on the LinB_B90A_ background and one synthetic variant each on the LinB_SS04-3_ (G1.7) and LinB_NM05_ (G1.8) background were made. One of the former (G1.3; T135L) was generated by mistake (physio-chemically similar T135A was intended) during gene synthesis. These eight generation one mutants were analyzed for β- and δ-HCH activities in order to determine the contributions of individual amino acid differences to the activity variation. Three LinB_B90A_ variants (G1.1–G1.3) showed ∼2 fold higher activity (Table 1), and these mutations (A83P, V134L and T135L), along with certain others, were used to make five second generation double and triple mutants on the LinB_B90A_ background. These mutants were also tested on β- and δ-HCH.

Overall eleven of the synthetic variants from both generations one and two yielded activity values within the range seen for the natural variants (0–214 and 0–8 min^−1^ for β- and δ-HCH respectively; Table 1). However, LinB_G2.3_ and LinB_G2.2_ yielded activities 2–4 and 30 fold, respectively, higher than any previously seen, and 4–8 and about 80 fold higher than those of the LinB_B90A_ reference variant (Table 1). β-HCH activities were again much higher than δ-HCH for all the synthetic variants and there was a strong correlation between β- and δ-HCH activities across the total of 23 natural and synthetic variants (r^2^ = 0.99; P<0.03).

Table 2 rearranges a subset of the data from Table 1 to show the effects of single amino acid differences at eight positions spread across the enzyme (T81A, A83P, V112A, V134L, T135L, L138I, H247S and I253M). In six of these cases the effects of each substitution could be examined in more than one haplotype background. All eight substitutions had significant effects in at least one, and generally, most of the backgrounds tested. For six of the substitutions the effect was 10 fold or greater. Notably however, all six substitutions tested in more than one background also showed significant, and often also large, effects of the background haplotype. The table shows clear cases of positive (V134L/T135L in LinB_G2.2_) and negative epistasis (A81 and P83 producing two-fold increases in β- and δ-HCH activities alone but a two-fold decrease together). Also a comparison of the A83P mutation in LinB_B90A_ and LinB_SS04-3_ illustrates the effect of the genetic background; this mutation doubled activity in the LinB_B90A_ background but decreased it by 10 fold in the LinB_SS04-3_ background.

**Table 2:** Pairwise comparisons of β- and δ-HCH data for variants characterized herein which differ by single amino acid changes.

Residues	Mutants	Haplotype																Turnover number (min^−1^)	
		2	6	7	81	83	112	134	135	138	147	150	222	223	224	247	253	β-HCH	δ-HCH
		S	K	P	T	A	V	V	T	L	D	L	A	I	A	H	I		
**T81A**	**W1.1/W1.3**	.	.	.	**T/A**	.	.	.	.	.	.	.	.	.	.	.	.	90±1/154±31	4±1/5±1
**T81A**	**G2.2/G2.3**	.	.	.	**T/A**	.	.	L	L	.	.	.	.	.	.	.	.	6920±272/422±12	241±6/31±6
**T81A**	**G2.5/G2.1**	.	.	.	**T/A**	P	.	.	.	.	.	.	.	.	.	.	.	4±1/44±6	2±1/4±1
**A83P**	**W1.1/G1.1**	.	.	.	.	**A/P**	.	.	.	.	.	.	.	.	.	.	.	90±1/182±17	4±1/6±1
**A83P**	**W1.3/G2.1**	.	.	.	A	**A/P**	.	.	.	.	.	.	.	.	.	.	.	154±31/44±6	5±1/4±1
**A83P**	**W1.7/W1.8**	.	.	.	.	**A/P**	A	L	A	I	.	.	.	.	.	S	M	0/214±13	0/6±1
**A83P**	**W1.4/G1.7**	.	.	.	A	**A/P**	.	.	.	.	.	.	.	.	.	S	.	170±15/17±2	8±1/0
**V112A**	**W1.10/W1.9**	.	.	.	A	.	**V/A**	L	A	I	.	.	.	.	.	S	.	74±7/177±9	4±1/3±1
**V134L**	**W1.1/G1.2**	.	.	.	.	.	.	**V/L**	.	.	.	.	.	.	.	.	.	90±1/138±10	4±1/9±2
**V134L**	**G1.3/G2.2**	.	.	.	.	.	.	**V/L**	L	.	.	.	.	.	.	.	.	194±3/6920±272	10±2/241±6
**T135L**	**W1.1/G1.3**	.	.	.	.	.	.	.	**T/L**	.	.	.	.	.	.	.	.	90±1/194±3	4±1/10±2
**T135L**	**G1.2/G2.2**	.	.	.	.	.	.	L	**T/L**	.	.	.	.	.	.	.	.	138±10/6920±272	9±2/241±6
**L138I**	**W1.1/G1.4**	.	.	.	.	.	.	.	.	**L/I**	.	.	.	.	.	.	.	90±1/9±1	4±1/11±2
**H247S**	**W1.1/G1.5**	.	.	.	.	.	.	.	.	.	.	.	.	.	.	**H/S**	.	90±1/1±1	4±1/0
**H247S**	**W1.3/W1.4**	.	.	.	A	.	.	.	.	.	.	.	.	.	.	**H/S**	.	154±31/170±15	5±1/8±1
**H247S**	**G2.1/G1.7**	.	.	.	A	P	.	.	.	.	.	.	.	.	.	**H/S**	**.**	44±6/17±2	4±1/0
**I253M**	**W1.1/G1.6**	.	.	.	.	.	.	.	.	.	.	.	.	.	.	.	**I/M**	90±1/20±1	4±1/0

The activity data are taken from Table 1 and abbreviated variant names are also as in Table 1. Haplotypes are also shown as per Table 1 (with LinB_B90A_ again used as the reference), except that residues that did not differ among the subset of variants included in these comparisons have been omitted. Blue and red colours are used to show the residues and activities corresponding to each variant in the pairwise comparisons.

The fact that the use of chaperones was necessary for expression of a few variants with higher activity suggests that some of the mutations nevertheless were deleterious for protein solubility or stability. We are now exploring whether the chaperone dependency of these variants could be ameliorated by stabilizing mutations.

**Figure 1:**
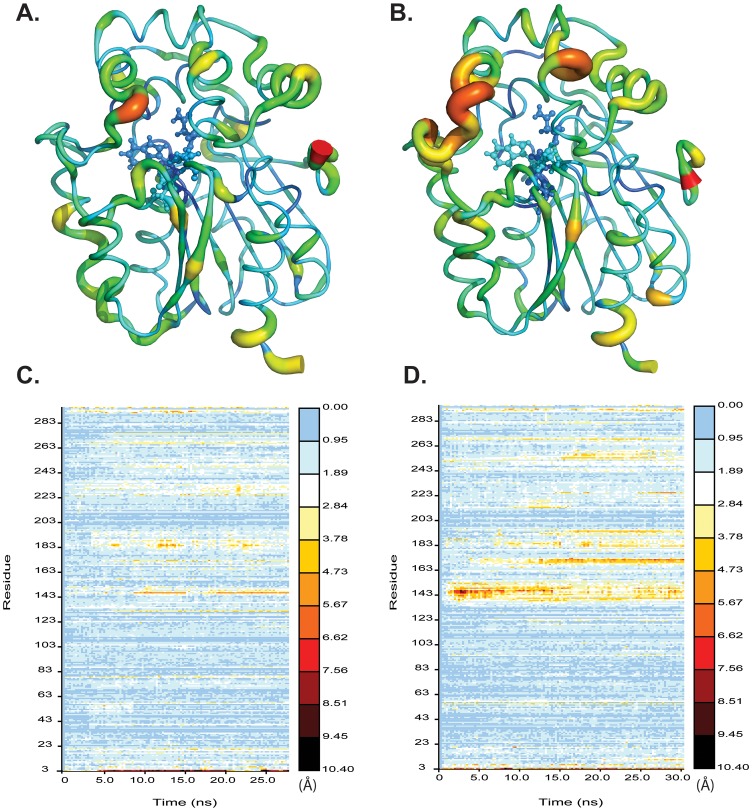
Comparative conformational flexibility within the cap domain of two LinB variants. Sausage diagram for LinB_B90A_ (A) and LinB_G2.2_ (B) in which the thickness of the chain as well as the colour denotes the average RMSD of each residue. The active site catalytic residues are rendered in ball and stick. The RMSD of each residue as a function of time is plotted for LinB_B90A_ (C) and LinB_G2.2_ (D).

Molecular dynamics simulations were used to probe the conformational dynamics of the synthetic LinB_G2.2_ variant relative to LinB_B90A_ (our reference variant). These simulations revealed large differences in conformational flexibility within the cap domain (Figure 1). This domain occludes the active site and has previously been shown to be more flexible in LinB relative to the related dehalogenases DhaA and DhlA [28]. Here we see that LinB_G2.2_ shows greatly enhanced flexibility in this region when compared to LinB_B90A_. Unlike some of the other variants of this enzyme, LinB_B90A_ has a histidine at position 247. This residue has been hypothesized to enhance activity by reducing solvent access to the active site [13]. Although this mechanism may be feasible for some of the smaller haloalkane dehalogenase substrates (such as dichloroethane and trichloropropane), HCH cannot easily diffuse into the partially occluded active site in LinB, nor can it easily enter through the side tunnels previously characterized for this enzyme [13]. As such, it seems reasonable to assume that increased flexibility in the cap domain plays an important role in the enhancement of catalysis as observed in LinB_G2.2_ relative to LinB_B90A_ by removing kinetic barriers to substrate binding (in spite of the fact that this potentially allows greater solvent penetration into the active site).

**Figure 2:**
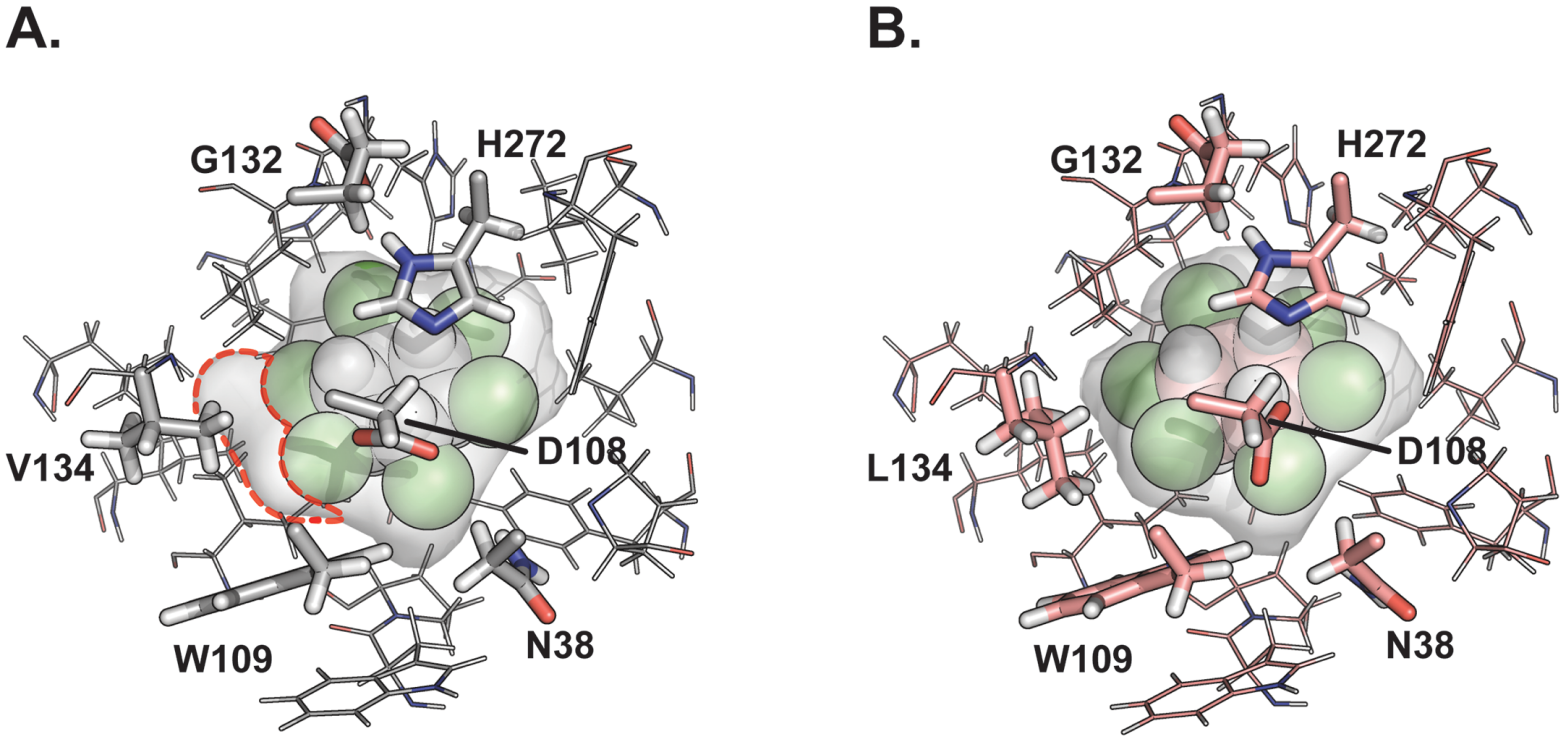
Approximate affinity of LinB_B90A_ and LinB_G2.2_ for β-HCH using Rosetta Ligand Dock program. Highest scoring docked pose for LinB_B90A_ (A) and LinB_G2.2_ (B). The ligand β-HCH is shown as Van der Waals spheres, catalytic residues (as well as residue 134 which differs between LinB_B90A_ and LinB_G2.2_) are rendered as thick sticks. Other residues within 5 Å of β-HCH are rendered as thin sticks. The empty space in the binding pocket of LinB_B90A_ that is filled by the V134L mutation of LinB_G2.2_ is outlined with red dashes.

**Figure 3:**
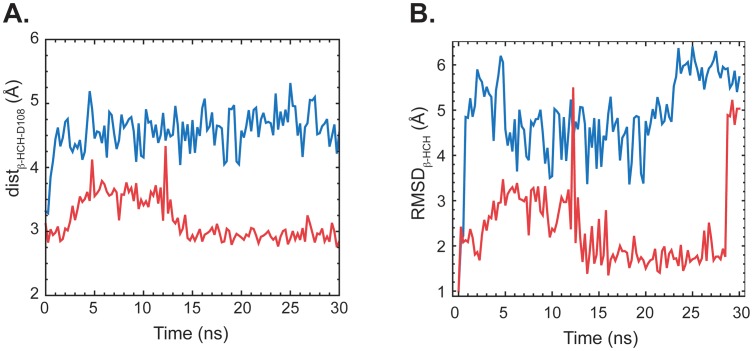
Molecular dynamics simulations of LinB with β-HCH bound in the active site. The distance between the nucleophilic oxygen of Asp108 and the center of mass of β-HCH is shown as a function of time (LinB_B90A_ in blue and LinB_G2.2_ in red) in panel A. Panel B shows the RMSD from the near-attack conformation for β-HCH as a function of time (LinB_B90A_ in blue and LinB_G2.2_ in red) in panel B.

Additionally, our simulations indicate that LinB_G2.2_ is able to bind β-HCH more tightly than LinB_B90A_. The average binding energy of β-HCH to LinB_G2.2_ was calculated to be −4.25±0.13 REU (Rosetta energy units) compared to that of LinB_B90A_, which was −2.54±0.22. This modest increase in affinity arises in part from more favourable hydrophobic interactions between L134 and β-HCH (Figure 2). This is also supported by results from molecular dynamics simulations with β-HCH bound, which show that LinB_G2.2_ is able to keep the ligand much closer to the catalytic conformation than LinB_B90A_ (Figure 3). Both the distance between the nucleophilic oxygen and the ligand centre of mass as well as the RMSD from the catalytic conformation are much lower for LinB_G2.2_ relative to LinB_B90A_ (Figure 3).

Collectively, these results imply that the V134L/T135L mutations of LinB_G2.2_ enhance catalysis by affecting the conformational plasticity of the cap domain and by holding the substrate in a suitable conformation for catalysis. More extensive simulations as well as additional biophysical and biochemical analysis are now needed to provide a more complete picture of the relevant conformational motions of this enzyme.

## Supporting Information

Figure S1 **Superposition of the ground state β-HCH structure and the β-HCH transition state as calculated by ab initio electronic structure theory.**
(DOCX)

Table S1 **Enzyme variants and primers.**
(DOCX)

Table S2 **Codon optimized gene sequences.**
(DOCX)
