# Supplementary material for: Kinetic and Sequence-Structure-Function Analysis of LinB Enzyme Variants with β- and δ-Hexachlorocyclohexane
Source: PLoS One. 2014 Jul 30;9(7):e103632. doi: 10.1371/journal.pone.0103632 (PMC4116220; doi:10.1371/journal.pone.0103632)
Supplement: Figure S1 — Superposition of the ground state β-HCH structure and the β-HCH transition state as calculated by ab initio electronic structure theory. (DOCX) [file pone.0103632.s001.docx]

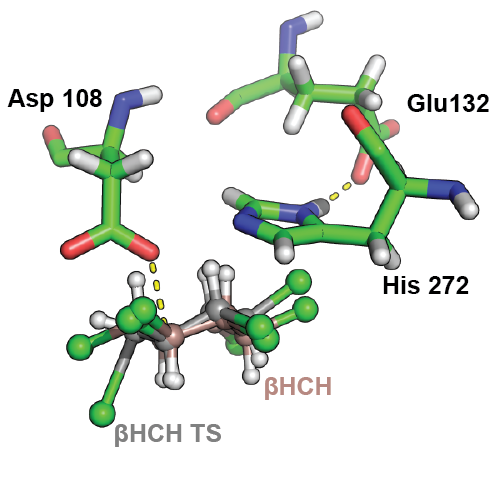


**Figure S1:** **Superposition of the ground state β-HCH structure and the β-HCH transition state as calculated by ab initio electronic structure theory**. The elongated carbon-chlorine bond can be seen in the transition state structure as well as the distance between the nucleophillic oxygen in Asp 108 and the electrophilic carbon. The other components of the catalytic triad (His 272 and Glu 132) are also shown. By fitting the ground state β-HCH structure onto the transition state and then minimizing the energy a catalytically favourable enzyme-substrate complex is obtained.
