# Supplementary material for: Kinetic and Sequence-Structure-Function Analysis of LinB Enzyme Variants with β- and δ-Hexachlorocyclohexane
Source: PLoS One. 2014 Jul 30;9(7):e103632. doi: 10.1371/journal.pone.0103632 (PMC4116220; doi:10.1371/journal.pone.0103632)
Supplement: Table S1 — Enzyme variants and primers. (DOCX) [file pone.0103632.s002.docx]

**Table S2. Codon optimized gene sequences**

| >W1.1_SS04-1 |
| --- |
| atgagcctgggcgcgaaaccgtttggcgaaaaaaaattcatcgaaatcaaaggccgtcgtatggcgtatattgatgaaggcaccggcgatccgattctgtttcagcatggcaatccgaccagcagctatctgtggcgtaacattatgccgcattgcgcgggtctgggccgtctgattgcgtgcgatctgattggcatgggcgatagcgataaactggacccgagcggtccggaacgttatgcgtatgcggaacatcgtgattatctggatgcgctgtgggaagcactggatctgggcgatcgtgtggtgctggttgtgcatgattggggctctgtgctgggctttgattgggcgcgtcgccatcgtgaacgtgtgcagggcattgcgtatatggaagcgctggccatgccgattgaatgggcggattttccggaacaggatcgtgacctgtttcaggcgtttcgtagccaggcgggcgaagaactggtgctgcaggataacgtgtttgtggaacaggtgctgccgggcctgattctgcgtccgctgtctgaagcggaaatggcggcgtatcgtgaaccgtttctggccgcgggtgaagcgcgtcgtccgaccctgagctggccgcgtcagattccgattgcgggcaccccggcggatgttgtggcgattgcgcgtgattatgcgggctggctgtctgaaagcccgattccgaaactgtttattaacgcggaaccgggcagcctgaccaccggccgtattcgtgatttttgccgtacctggccgaaccagaccgaaattaccgtggcgggtgcgcattttattcaggaagatagcccggacgaaattggtgcggcgattgcggcgtttgtgcgtcgtctgcgtccggcgtaatga |
|  |
| >W1.9_SS04-2 |
| atgagcctgggcgcgaaaccgtttggcgaaaaaaaattcatcgaaatcaaaggccgtcgtatggcgtatattgatgaaggcaccggcgatccgattctgtttcagcatggcaatccgaccagcagctatctgtggcgtaacattatgccgcattgcgcgggtctgggccgtctgattgcgtgcgatctgattggcatgggcgatagcgataaactggacccgagcggtccggaacgttatgcgtatgcggaacaccgtgattatctggatgcgctgtgggaagcgctggatctgggcgatcgtgtggtgctggttgtgcatgattggggcagcgtgctgggctttgattgggcgcgtcgtcatcgtgaacgtgtgcagggcattgcgtatatggaagcggtgaccatgccgctggaatgggcggattttccggaacaggatcgtgacctgtttcaggcgtttcgtagccaggcgggcgaagaactggtgctgcaggataacgtgtttgtggaacaggtgctgccgggcctgattctgcgtccgctgtctgaagcggaaatggcggcgtatcgtgaaccgtttctggccgcgggtgaagcgcgtcgtccgaccctgagctggccgcgtcagattccgattgcgggcactccggcggatgttgtggcgattgcgcgtgattatgcgggctggctgtctgaaagcccgattccgaaactgtttattaacgcggaaccgggcagcctgaccaccggccgtattcgtgatttttgccgtacctggccgaaccagaccgaaattaccgtggcgggtgcgcattttattcaggaagatagcccggacgaaattggtgcggcgattgcggcgtttgtgcgtcgtctgcgtccggcgtaatga |
|  |
| >W1.8_SS04-5 |
| atgagcctgggcgcgaaaccgtttggcgaaaaaaaattcatcgaaatcaaaggccgtcgtatggcgtatattgatgaaggcaccggcgatccgattctgtttcagcatggcaatccgaccagcagctatctgtggcgtaacattatgccgcattgcgcgggtctgggccgtctgattgcgtgcgatctgattggcatgggcgatagcgataaactggacccgagcggtccggaacgttatacctatccggaacatcgtgattatctggatgcgctgtgggaagcactggatctgggcgatcgtgtggtgctggttgtgcatgattggggctctgcgctgggctttgattgggcgcgtcgccatcgtgaacgtgtgcagggcattgcgtatatggaagcgctggccatgccgattgaatgggcggattttccggaacaggatcgtgacctgtttcaggcgtttcgtagccaggcgggcgaagaactggtgctgcaggataacgtgtttgtggaacaggtgctgccgggcctgattctgcgtccgctgtctgaagcggaaatggcggcgtatcgtgaaccgtttctggccgcgggtgaagcgcgtcgtccgaccctgagctggccgcgtcagattccgattgcgggcaccccggcggatgttgtggcgattgcgcgtgattatgcgggctggctgtctgaaagcccgattccgaaactgtttattaacgcggaaccgggcagcctgaccaccggccgtatgcgtgatttttgccgtacctggccgaaccagaccgaaattaccgtggcgggtgcgcattttattcaggaagatagcccggacgaaattggtgcggcgattgcggcgtttgtgcgtcgtctgcgtccggcgtaatga |
|  |
| >W1.7_Sp+ |
| atgagcctgggcgcgaaaccgtttggcgaaaaaaaattcatcgaaatcaaaggccgtcgtatggcgtatattgatgaaggcaccggcgatccgattctgtttcagcatggcaatccgaccagcagctatctgtggcgtaacattatgccgcattgcgcgggtctgggccgtctgattgcgtgcgatctgattggcatgggcgatagcgataaactggacccgagcggtccggaacgttatacctatgcggaacatcgtgattatctggatgcgctgtgggaagcactggatctgggcgatcgtgtggtgctggttgtgcatgattggggctctgcgctgggctttgattgggcgcgtcgccatcgtgaacgtgtgcagggcattgcgtatatggaagcgctggccatgccgattgaatgggcggattttccggaacaggatcgtgacctgtttcaggcgtttcgtagccaggcgggcgaagaactggtgctgcaggataacgtgtttgtggaacaggtgctgccgggcctgattctgcgtccgctgtctgaagcggaaatggcggcgtatcgtgaaccgtttctggccgcgggtgaagcgcgtcgtccgaccctgagctggccgcgtcagattccgattgcgggcaccccggcggatgttgtggcgattgcgcgtgattatgcgggctggctgtctgaaagcccgattccgaaactgtttattaacgcggaaccgggcagcctgaccaccggccgtatgcgtgatttttgccgtacctggccgaaccagaccgaaattaccgtggcgggtgcgcattttattcaggaagatagcccggacgaaattggtgcggcgattgcggcgtttgtgcgtcgtctgcgtccggcgtaatga |
|  |
| >W1.6_UT26 |
| atgagcctgggcgcgaaaccgtttggcgaaaaaaaattcatcgaaatcaaaggccgtcgtatggcgtatattgatgaaggcaccggcgatccgattctgtttcagcatggcaatccgaccagcagctatctgtggcgtaacattatgccgcattgcgcgggtctgggccgtctgattgcgtgcgatctgattggcatgggcgatagcgataaactggacccgagcggtccggaacgttatgcgtatgcggaacatcgtgattatctggatgcgctgtgggaagcactggatctgggcgatcgtgtggtgctggttgtgcatgattggggctctgcgctgggctttgattgggcgcgtcgccatcgtgaacgtgtgcagggcattgcgtatatggaagcgattgccatgccgattgaatgggcggattttccggaacaggatcgtgacctgtttcaggcgtttcgtagccaggcgggcgaagaactggtgctgcaggataacgtgtttgtggaacaggtgctgccgggcctgattctgcgtccgctgtctgaagcggaaatggcggcgtatcgtgaaccgtttctggccgcgggtgaagcgcgtcgtccgaccctgagctggccgcgtcagattccgattgcgggcaccccggcggatgttgtggcgattgcgcgtgattatgcgggctggctgtctgaaagcccgattccgaaactgtttattaacgcggaaccgggcgcgctgaccaccggccgtatgcgtgatttttgccgtacctggccgaaccagaccgaaattaccgtggcgggtgcgcattttattcaggaagatagcccggacgaaattggtgcggcgattgcggcgtttgtgcgtcgtctgcgtccggcgtaatga |
|  |
| >W1.5_NM05 |
| atgattctgggcgcgaacgcgtttggcgaaaaaaaattcatcgaaatcaaaggccgtcgtatggcgtatattgatgaaggcaccggcgatccgattctgtttcagcatggcaatccgaccagcagctatctgtggcgtaacattatgccgcattgcgcgggtctgggccgtctgattgcgtgcgatctgattggcatgggcgatagcgataaactggacccgagcggtccggaacgttatgcgtatgcggaacatcgtgattatctggatgcgctgtgggaagcgctggatctgggcgatcgtgtggtgctggttgtgcatgattggggcagcgtgctgggctttgattgggcgcgtcgtcatcgtgaacgtgtgcagggcattgcgtatatggaagcggtgaccatgccgctggaatgggcggattttccggaacagtatcgcgatatgtttcaggcgtttcgtagccaggcgggcgaagaactggtgctgcaggataacgtgtttgtggaacaggtgctgccgggcctgattctgcgtccgctgtctgaagcggaaatggcggcgtatcgtgaaccgtttctggccgcgggtgaagcgcgtcgtccgaccctgagctggccgcgtcagattccgattgcgggcaccccggcggatgtggtggtgcatgcgcgtgattatgcgggctggctgtctgaaagcccgattccgaaactgtttattaacgcggaaccgggtgcgctgaccaccggccgtatgcgtgatttttgccgtacctggccgaaccagaccgaaattaccgtggcgggtgcgcattttattcaggaagatagcccggacgaaattggtgcggcgattgcggcgtttgtgcgtcgtctgcgtccggcgtaatga |
|  |
| >W1.4_SS04-3 |
| atgagcctgggcgcgaaaccgtttggcgaaaaaaaattcatcgaaatcaaaggccgtcgtatggcgtatattgatgaaggcaccggcgatccgattctgtttcagcatggcaatccgaccagcagctatctgtggcgtaacattatgccgcattgcgcgggtctgggccgtctgattgcgtgcgatctgattggcatgggcgatagcgataaactggacccgagcggtccggaacgttatgcgtatgcggaacaccgtgattatctggatgcgctgtgggaagcgctggatctgggcgatcgtgtggtgctggttgtgcatgattggggcagcgtgctgggctttgattgggcgcgtcgtcatcgtgaacgtgtgcagggcattgcgtatatggaagcggtgaccatgccgctggaatgggcggattttccggaacaggatcgtgacctgtttcaggcgtttcgtagccaggcgggcgaagaactggtgctgcaggataacgtgtttgtggaacaggtgctgccgggcctgattctgcgtccgctgtctgaagcggaaatggcggcgtatcgtgaaccgtttctggccgcgggtgaagcgcgtcgtccgaccctgagctggccgcgtcagattccgattgcgggcactccggcggatgttgtggcgattgcgcgtgattatgcgggctggctgtctgaaagcccgattccgaaactgtttattaacgcggaaccgggcagcctgaccaccggccgtattcgtgatttttgccgtacctggccgaaccagaccgaaattaccgtggcgggtgcgcattttattcaggaagatagcccggacgaaattggtgcggcgattgcggcgtttgtgcgtcgtctgcgtccggcgtaatga |
|  |
| >W1.3_ITRC-5-A |
| atgagcctgggcgcgaaaccgtttggcgaaaaaaaattcatcgaaatcaaaggccgtcgtatggcgtatattgatgaaggcaccggcgatccgattctgtttcagcatggcaatccgaccagcagctatctgtggcgtaacattatgccgcattgcgcgggtctgggccgtctgattgcgtgcgatctgattggcatgggcgatagcgataaactggacccgagcggtccggaacgttatgcgtatgcggaacaccgtgattatctggatgcgctgtgggaagcgctggatctgggcgatcgtgtggtgctggttgtgcatgattggggcagcgtgctgggctttgattgggcgcgtcgtcatcgtgaacgtgtgcagggcattgcgtatatggaagcggtgaccatgccgctggaatgggcggattttccggaacaggatcgtgacctgtttcaggcgtttcgtagccaggcgggcgaagaactggtgctgcaggataacgtgtttgtggaacaggtgctgccgggcctgattctgcgtccgctgtctgaagcggaaatggcggcgtatcgtgaaccgtttctggccgcgggtgaagcgcgtcgtccgaccctgagctggccgcgtcagattccgattgcgggcactccggcggatgttgtggcgattgtgcgtgattatgcgggctggctgtctgaaagcccgattccgaaactgtttattaacgcggaaccgggcagcctgaccaccggccgtattcgtgatttttgccgtacctggccgaaccagaccgaaattaccgtggcgggtgcgcattttattcaggaagatagcccggacgaaattggtgcggcgattgcggcgtttgtgcgtcgtctgcgtccggcgtaatga |
|  |
| >W1.2_ITRC-5-B |
| atgagcctgggcgcgaaaccgtttggcgaaaaaaaattcatcgaaatcaaaggccgtcgtatggcgtatattgatgaaggcaccggcgatccgattctgtttcagcatggcaatccgaccagcagctatctgtggcgtaacattatgccgcattgcgcgggtctgggccgtctgattgcgtgcgatctgattggcatgggcgatagcgataaactggacccgagcggtccggaacgttatgcgtatgcggaacaccgtgattatctggatgcgctgtgggaagcgctggatctgggcgatcgtgtggtgctggttgtgcatgattggggcagcgtgctgggctttgattgggcgcgtcgtcatcgtgaacgtgtgcagggcattgcgtatatggaagcggtgaccatgccgctggaatgggcggattttccggaacaggatcgtgacctgtttcaggcgtttcgtagccaggcgggcgaagaactggtgctgcaggataacgtgtttgtggaacaggtgctgccgggcctgattctgcgtccgctgtctgaagcggaaatggcggcgtatcgtgaaccgtttctggccgcgggtgaagcgcgtcgtccgaccctgagctggccgcgtcagattccgattgcgggcactccggcggatgttgtggcgattgcgcgtgattatgcgggctggctgtctgaaagcccgattccgaaactgtttattaacgcggaaccgggccatctgaccaccggccgtattcgtgatttttgccgtacctggccgaaccagaccgaaattaccgtggcgggtgcgcattttattcaggaagatagcccggacgaaattggtgcggcgattgcggcgtttgtgcgtcgtctgcgtccggcgtaatga |
|  |
| >W1.1_B90A |
| atgagcctgggcgcgaaaccgtttggcgaaaaaaaattcatcgaaatcaaaggccgtcgtatggcgtatattgatgaaggcaccggcgatccgattctgtttcagcatggcaatccgaccagcagctatctgtggcgtaacattatgccgcattgcgcgggtctgggccgtctgattgcgtgcgatctgattggcatgggcgatagcgataaactggacccgagcggtccggaacgttatacctatgcggaacaccgtgattatctggatgcgctgtgggaagcgctggatctgggcgatcgtgtggtgctggttgtgcatgattggggcagcgtgctgggctttgattgggcgcgtcgtcatcgtgaacgtgtgcagggcattgcgtatatggaagcggtgaccatgccgctggaatgggcggattttccggaacaggatcgtgacctgtttcaggcgtttcgtagccaggcgggcgaagaactggtgctgcaggataacgtgtttgtggaacaggtgctgccgggcctgattctgcgtccgctgtctgaagcggaaatggcggcgtatcgtgaaccgtttctggccgcgggtgaagcgcgtcgtccgaccctgagctggccgcgtcagattccgattgcgggcactccggcggatgttgtggcgattgcgcgtgattatgcgggctggctgtctgaaagcccgattccgaaactgtttattaacgcggaaccgggccatctgaccaccggccgtattcgtgatttttgccgtacctggccgaaccagaccgaaattaccgtggcgggtgcgcattttattcaggaagatagcccggacgaaattggtgcggcgattgcggcgtttgtgcgtcgtctgcgtccggcgtaatga |
|  |
| >G1.1 |
| atgagcctgggtgcaaaaccgtttggcgaaaaaaaatttattgaaattaaaggccgtcgcatggcatatattgatgaaggcaccggtgatccgattctgtttcagcatggtaatccgaccagcagctatctgtggcgtaatattatgccgcattgtgcaggtctgggtcgtctgattgcatgtgatctgattggtatgggcgatagcgataaactggatccgagcggtccggaacgttatacctatccggaacatcgtgattatctggatgcactgtgggaagcactggatctgggtgatcgtgttgttctggttgttcatgattggggtagcgttctgggttttgattgggcacgtcgtcatcgtgaacgtgttcagggtattgcatatatggaagcagttaccatgccgctggaatgggcagattttccggaacaggatcgtgacctgtttcaggcatttcgtagccaggcaggcgaagaactggttctgcaggataatgtttttgttgaacaggttctgcctggtctgattctgcgtccgctgagcgaagcagaaatggcagcatatcgtgaaccgtttctggcagccggtgaagcacgtcgtccgaccctgtcttggcctcgtcagattccgattgcaggtacaccggcagatgttgttgcaattgcacgtgattatgcaggttggctgagcgaaagcccgattccgaaactgtttattaatgccgaaccgggtcatctgaccaccggtcgtattcgtgatttttgtcgtacctggccgaatcagaccgaaattaccgttgccggtgcacattttattcaggaagattctccggacgaaattggtgcagcaattgcagcatttgttcgtcgtctgcgtccggcataatga |
|  |
| >G1.2 |
| atgagcctgggtgcaaaaccgtttggcgaaaaaaaatttattgaaattaaaggccgtcgcatggcatatattgatgaaggcaccggtgatccgattctgtttcagcatggtaatccgaccagcagctatctgtggcgtaatattatgccgcattgtgcaggtctgggtcgtctgattgcatgtgatctgattggtatgggcgatagcgataaactggatccgagcggtccggaacgttatacctatgctgaacatcgtgattatctggatgcactgtgggaagcactggatctgggtgatcgtgttgttctggttgttcatgattggggtagcgttctgggttttgattgggcacgtcgtcatcgtgaacgtgttcagggtattgcatatatggaagcactgaccatgccgctggaatgggcagattttccggaacaggatcgtgacctgtttcaggcatttcgtagccaggcaggcgaagaactggttctgcaggataatgtttttgttgaacaggttctgcctggtctgattctgcgtccgctgagcgaagcagaaatggcagcatatcgtgaaccgtttctggcagccggtgaagcacgtcgtccgaccctgtcttggcctcgtcagattccgattgcaggtacaccggcagatgttgttgcaattgcacgtgattatgcaggttggctgagcgaaagcccgattccgaaactgtttattaatgccgaaccgggtcatctgaccaccggtcgtattcgtgatttttgtcgtacctggccgaatcagaccgaaattaccgttgccggtgcacattttattcaggaagattctccggacgaaattggtgcagcaattgcagcatttgttcgtcgtctgcgtccggcataatga |
|  |
| >G1.3 |
| atgagcctgggtgcaaaaccgtttggcgaaaaaaaatttattgaaattaaaggccgtcgcatggcatatattgatgaaggcaccggtgatccgattctgtttcagcatggtaatccgaccagcagctatctgtggcgtaatattatgccgcattgtgcaggtctgggtcgtctgattgcatgtgatctgattggtatgggcgatagcgataaactggatccgagcggtccggaacgttatacctatgctgaacatcgtgattatctggatgcactgtgggaagcactggatctgggtgatcgtgttgttctggttgttcatgattggggtagcgttctgggttttgattgggcacgtcgtcatcgtgaacgtgttcagggtattgcatatatggaagcagttctgatgccgctggaatgggcagattttccggaacaggatcgtgacctgtttcaggcatttcgtagccaggcaggcgaagaactggttctgcaggataatgtttttgttgaacaggttctgcctggtctgattctgcgtccgctgagcgaagcagaaatggcagcatatcgtgaaccgtttctggcagccggtgaagcacgtcgtccgaccctgtcttggcctcgtcagattccgattgcaggtacaccggcagatgttgttgcaattgcacgtgattatgcaggttggctgagcgaaagcccgattccgaaactgtttattaatgccgaaccgggtcatctgaccaccggtcgtattcgtgatttttgtcgtacctggccgaatcagaccgaaattaccgttgccggtgcacattttattcaggaagattctccggacgaaattggtgcagcaattgcagcatttgttcgtcgtctgcgtccggcataatga |
|  |
| >G1.4 |
| atgagcctgggtgcaaaaccgtttggcgaaaaaaaatttattgaaattaaaggccgtcgcatggcatatattgatgaaggcaccggtgatccgattctgtttcagcatggtaatccgaccagcagctatctgtggcgtaatattatgccgcattgtgcaggtctgggtcgtctgattgcatgtgatctgattggtatgggcgatagcgataaactggatccgagcggtccggaacgttatacctatgctgaacatcgtgattatctggatgcactgtgggaagcactggatctgggtgatcgtgttgttctggttgttcatgattggggtagcgttctgggttttgattgggcacgtcgtcatcgtgaacgtgttcagggtattgcatatatggaagcagttaccatgccgattgaatgggcagattttccggaacaggatcgtgacctgtttcaggcatttcgtagccaggcaggcgaagaactggttctgcaggataatgtttttgttgaacaggttctgcctggtctgattctgcgtccgctgagcgaagcagaaatggcagcatatcgtgaaccgtttctggcagccggtgaagcacgtcgtccgaccctgtcttggcctcgtcagattccgattgcaggtacaccggcagatgttgttgcaattgcacgtgattatgcaggttggctgagcgaaagcccgattccgaaactgtttattaatgccgaaccgggtcatctgaccaccggtcgtattcgtgatttttgtcgtacctggccgaatcagaccgaaattaccgttgccggtgcacattttattcaggaagattctccggacgaaattggtgcagcaattgcagcatttgttcgtcgtctgcgtccggcataatga |
|  |
| >G1.5 |
| atgagcctgggtgcaaaaccgtttggcgaaaaaaaatttattgaaattaaaggccgtcgcatggcatatattgatgaaggcaccggtgatccgattctgtttcagcatggtaatccgaccagcagctatctgtggcgtaatattatgccgcattgtgcaggtctgggtcgtctgattgcatgtgatctgattggtatgggcgatagcgataaactggatccgagcggtccggaacgttatacctatgctgaacatcgtgattatctggatgcactgtgggaagcactggatctgggtgatcgtgttgttctggttgttcatgattggggtagcgttctgggttttgattgggcacgtcgtcatcgtgaacgtgttcagggtattgcatatatggaagcagttaccatgccgctggaatgggcagattttccggaacaggatcgtgacctgtttcaggcatttcgtagccaggcaggcgaagaactggttctgcaggataatgtttttgttgaacaggttctgcctggtctgattctgcgtccgctgagcgaagcagaaatggcagcatatcgtgaaccgtttctggcagccggtgaagcacgtcgtccgaccctgtcttggcctcgtcagattccgattgcaggtacaccggcagatgttgttgcaattgcacgtgattatgcaggttggctgagcgaaagcccgattccgaaactgtttattaatgccgaaccgggtagcctgaccaccggtcgtattcgtgatttttgtcgtacctggccgaatcagaccgaaattaccgttgccggtgcacattttattcaggaagattctccggacgaaattggtgcagcaattgcagcatttgttcgtcgtctgcgtccggcataatga |
|  |
| >G1.6 |
| atgagcctgggtgcaaaaccgtttggcgaaaaaaaatttattgaaattaaaggccgtcgcatggcatatattgatgaaggcaccggtgatccgattctgtttcagcatggtaatccgaccagcagctatctgtggcgtaatattatgccgcattgtgcaggtctgggtcgtctgattgcatgtgatctgattggtatgggcgatagcgataaactggatccgagcggtccggaacgttatacctatgctgaacatcgtgattatctggatgcactgtgggaagcactggatctgggtgatcgtgttgttctggttgttcatgattggggtagcgttctgggttttgattgggcacgtcgtcatcgtgaacgtgttcagggtattgcatatatggaagcagttaccatgccgctggaatgggcagattttccggaacaggatcgtgacctgtttcaggcatttcgtagccaggcaggcgaagaactggttctgcaggataatgtttttgttgaacaggttctgcctggtctgattctgcgtccgctgagcgaagcagaaatggcagcatatcgtgaaccgtttctggcagccggtgaagcacgtcgtccgaccctgtcttggcctcgtcagattccgattgcaggtacaccggcagatgttgttgcaattgcacgtgattatgcaggttggctgagcgaaagcccgattccgaaactgtttattaatgccgaaccgggtcatctgaccaccggtcgtatgcgtgatttttgtcgtacctggccgaatcagaccgaaattaccgttgccggtgcacattttattcaggaagattctccggacgaaattggtgcagcaattgcagcatttgttcgtcgtctgcgtccggcataatga |
|  |
| >G1.7 |
| atgagcctgggtgcaaaaccgtttggcgaaaaaaaatttattgaaattaaaggccgtcgcatggcatatattgatgaaggcaccggtgatccgattctgtttcagcatggtaatccgaccagcagctatctgtggcgtaatattatgccgcattgtgcaggtctgggtcgtctgattgcatgtgatctgattggtatgggcgatagcgataaactggatccgagcggtccggaacgttatgcatatccggaacatcgtgattatctggatgcactgtgggaagcactggatctgggtgatcgtgttgttctggttgttcatgattggggtagcgttctgggttttgattgggcacgtcgtcatcgtgaacgtgttcagggtattgcatatatggaagcagttaccatgccgctggaatgggcagattttccggaacaggatcgtgacctgtttcaggcatttcgtagccaggcaggcgaagaactggttctgcaggataatgtttttgttgaacaggttctgcctggtctgattctgcgtccgctgagcgaagcagaaatggcagcatatcgtgaaccgtttctggcagccggtgaagcacgtcgtccgaccctgtcttggcctcgtcagattccgattgcaggtacaccggcagatgttgttgcaattgcacgtgattatgcaggttggctgagcgaaagcccgattccgaaactgtttattaatgccgaaccgggtagcctgaccaccggtcgtattcgtgatttttgtcgtacctggccgaatcagaccgaaattaccgttgccggtgcacattttattcaggaagattctccggacgaaattggtgcagcaattgcagcatttgttcgtcgtctgcgtccggcataatga |
|  |
| >G1.8 |
| atgattctgggtgcaaatgcatttggcgaaaaaaaatttattgaaattaaaggccgtcgcatggcatatattgatgaaggcaccggtgatccgattctgtttcagcatggtaatccgaccagcagctatctgtggcgtaatattatgccgcattgtgcaggtctgggtcgtctgattgcatgtgatctgattggtatgggcgatagcgataaactggatccgagcggtccggaacgttatgcatatgctgaacatcgtgattatctggatgcactgtgggaagcactggatctgggtgatcgtgttgttctggttgttcatgattggggtagcgttctgggttttgattgggcacgtcgtcatcgtgaacgtgttcagggtattgcatatatggaagcagttaccatgccgctggaatgggcagattttccggaacagtatcgtgacatgtttcaggcatttcgtagccaggcaggcgaagaactggttctgcaggataatgtttttgttgaacaggttctgcctggtctgattctgcgtccgctgagcgaagcagaaatggcagcatatcgtgaaccgtttctggcagccggtgaagcacgtcgtccgaccctgtcttggcctcgtcagattccgattgcaggtacaccggcagatgttgttgttcatgcacgtgattatgcaggttggctgagcgaaagcccgattccgaaactgtttattaatgccgaaccgggtcatctgaccaccggtcgtatgcgtgatttttgtcgtacctggccgaatcagaccgaaattaccgttgccggtgcacattttattcaggaagattctccggacgaaattggtgcagcaattgcagcatttgttcgtcgtctgcgtccggcataatga |
|  |
| >G2.1 |
| atgagcctgggtgcaaaaccgtttggcgaaaaaaaatttattgaaattaaaggccgtcgcatggcatatattgatgaaggcaccggtgatccgattctgtttcagcatggtaatccgaccagcagctatctgtggcgtaatattatgccgcattgtgcaggtctgggtcgtctgattgcatgtgatctgattggtatgggcgatagcgataaactggatccgagcggtccggaacgttatgcatatccggaacatcgtgattatctggatgcactgtgggaagcactggatctgggtgatcgtgttgttctggttgttcatgattggggtagcgttctgggttttgattgggcacgtcgtcatcgtgaacgtgttcagggtattgcatatatggaagcagttaccatgccgctggaatgggcagattttccggaacaggatcgtgacctgtttcaggcatttcgtagccaggcaggcgaagaactggttctgcaggataatgtttttgttgaacaggttctgcctggtctgattctgcgtccgctgagcgaagcagaaatggcagcatatcgtgaaccgtttctggcagccggtgaagcacgtcgtccgaccctgtcttggcctcgtcagattccgattgcaggtacaccggcagatgttgttgcaattgcacgtgattatgcaggttggctgagcgaaagcccgattccgaaactgtttattaatgccgaaccgggtcatctgaccaccggtcgtattcgtgatttttgtcgtacctggccgaatcagaccgaaattaccgttgccggtgcacattttattcaggaagattctccggacgaaattggtgcagcaattgcagcatttgttcgtcgtctgcgtccggcataatga |
|  |
| >G2.2 |
| atgagcctgggtgcaaaaccgtttggcgaaaaaaaatttattgaaattaaaggccgtcgcatggcatatattgatgaaggcaccggtgatccgattctgtttcagcatggtaatccgaccagcagctatctgtggcgtaatattatgccgcattgtgcaggtctgggtcgtctgattgcatgtgatctgattggtatgggcgatagcgataaactggatccgagcggtccggaacgttatgcatatgcagaacatcgtgattatctggatgcactgtgggaagcactggatctgggtgatcgtgttgttctggttgttcatgattggggtagcgttctgggttttgattgggcacgtcgtcatcgtgaacgtgttcagggtattgcatatatggaagcactgctgatgccgctggaatgggcagattttccggaacaggatcgtgacctgtttcaggcatttcgtagccaggcaggcgaagaactggttctgcaggataatgtttttgttgaacaggttctgcctggtctgattctgcgtccgctgagcgaagcagaaatggcagcatatcgtgaaccgtttctggcagccggtgaagcacgtcgtccgaccctgtcttggcctcgtcagattccgattgcaggtacaccggcagatgttgttgcaattgcacgtgattatgcaggttggctgagcgaaagcccgattccgaaactgtttattaatgccgaaccgggtcatctgaccaccggtcgtattcgtgatttttgtcgtacctggccgaatcagaccgaaattaccgttgccggtgcacattttattcaggaagattctccggacgaaattggtgcagcaattgcagcatttgttcgtcgtctgcgtccggcataatga |
|  |
| >G2.3 |
| atgagcctgggtgcaaaaccgtttggcgaaaaaaaatttattgaaattaaaggccgtcgcatggcatatattgatgaaggcaccggtgatccgattctgtttcagcatggtaatccgaccagcagctatctgtggcgtaatattatgccgcattgtgcaggtctgggtcgtctgattgcatgtgatctgattggtatgggcgatagcgataaactggatccgagcggtccggaacgttatgcatatgcagaacatcgtgattatctggatgcactgtgggaagcactggatctgggtgatcgtgttgttctggttgttcatgattggggtagcgttctgggttttgattgggcacgtcgtcatcgtgaacgtgttcagggtattgcatatatggaagcactgctgatgccgctggaatgggcagattttccggaacaggatcgtgacctgtttcaggcatttcgtagccaggcaggcgaagaactggttctgcaggataatgtttttgttgaacaggttctgcctggtctgattctgcgtccgctgagcgaagcagaaatggcagcatatcgtgaaccgtttctggcagccggtgaagcacgtcgtccgaccctgtcttggcctcgtcagattccgattgcaggtacaccggcagatgttgttgcaattgcacgtgattatgcaggttggctgagcgaaagcccgattccgaaactgtttattaatgccgaaccgggtcatctgaccaccggtcgtattcgtgatttttgtcgtacctggccgaatcagaccgaaattaccgttgccggtgcacattttattcaggaagattctccggacgaaattggtgcagcaattgcagcatttgttcgtcgtctgcgtccggcataatga |
|  |
| >G2.4 |
| atgagcctgggtgcaaaaccgtttggcgaaaaaaaatttattgaaattaaaggccgtcgcatggcatatattgatgaaggcaccggtgatccgattctgtttcagcatggtaatccgaccagcagctatctgtggcgtaatattatgccgcattgtgcaggtctgggtcgtctgattgcatgtgatctgattggtatgggcgatagcgataaactggatccgagcggtccggaacgttatacctatgcagaacatcgtgattatctggatgcactgtgggaagcactggatctgggtgatcgtgttgttctggttgttcatgattggggtagcgttctgggttttgattgggcacgtcgtcatcgtgaacgtgttcagggtattgcatatatggaagcagttaccatgccgattgaatgggcagattttccggaacaggatcgtgacctgtttcaggcatttcgtagccaggcaggcgaagaactggttctgcaggataatgtttttgttgaacaggttctgcctggtctgattctgcgtccgctgagcgaagcagaaatggcagcatatcgtgaaccgtttctggcagccggtgaagcacgtcgtccgaccctgtcttggcctcgtcagattccgattgcaggtacaccggcagatgttgttgcaattgcacgtgattatgcaggttggctgagcgaaagcccgattccgaaactgtttattaatgccgaaccgggtagcctgaccaccggtcgtatgcgtgatttttgtcgtacctggccgaatcagaccgaaattaccgttgccggtgcacattttattcaggaagattctccggacgaaattggtgcagcaattgcagcatttgttcgtcgtctgcgtccggcataatga |
|  |
| >G2.5 |
| atgagcctgggtgcaaaaccgtttggcgaaaaaaaatttattgaaattaaaggccgtcgcatggcatatattgatgaaggcaccggtgatccgattctgtttcagcatggtaatccgaccagcagctatctgtggcgtaatattatgccgcattgtgcaggtctgggtcgtctgattgcatgtgatctgattggtatgggcgatagcgataaactggatccgagcggtccggaacgttatacctatccggaacatcgtgattatctggatgcactgtgggaagcactggatctgggtgatcgtgttgttctggttgttcatgattggggtagcgttctgggttttgattgggcacgtcgtcatcgtgaacgtgttcagggtattgcatatatggaagcagttaccatgccgctggaatgggcagattttccggaacaggatcgtgacctgtttcaggcatttcgtagccaggcaggcgaagaactggttctgcaggataatgtttttgttgaacaggttctgcctggtctgattctgcgtccgctgagcgaagcagaaatggcagcatatcgtgaaccgtttctggcagccggtgaagcacgtcgtccgaccctgtcttggcctcgtcagattccgattgcaggtacaccggcagatgttgttgcaattgcacgtgattatgcaggttggctgagcgaaagcccgattccgaaactgtttattaatgccgaaccgggtagcctgaccaccggtcgtattcgtgatttttgtcgtacctggccgaatcagaccgaaattaccgttgccggtgcacattttattcaggaagattctccggacgaaattggtgcagcaattgcagcatttgttcgtcgtctgcgtccggcataatga |
