# Supplementary material for: Kinetic and Sequence-Structure-Function Analysis of LinB Enzyme Variants with β- and δ-Hexachlorocyclohexane
Source: PLoS One. 2014 Jul 30;9(7):e103632. doi: 10.1371/journal.pone.0103632 (PMC4116220; doi:10.1371/journal.pone.0103632)
Supplement: Table S2 — Codon optimized gene sequences. (DOCX) [file pone.0103632.s003.docx]

**Table S1. Enzyme variants and primers**

| **Denotation** | **Strain (enzyme)** | **Primer set** | |
| --- | --- | --- | --- |
| W1.1 | *Sphingobium indicum* B90A (LinB_B90A_) | *CO-linB-F1* | *CO-linB-R1* |
| W1.2 | *Pseudomonas aeruginosa* ITRC-5 (LinB_ITRC-5-B_) |  |  |
| W1.3 | *Pseudomonas aeruginosa* ITRC-5 (LinB_ITRC-5-A_) |  |  |
| W1.4 | *Sphingomonas* sp. SS04-3 (LinB_SS04-3_) | *CO-linB-F2* | *CO-linB-R1* |
| W1.5 | *Sphingomonas* sp. NM05 (LinB_NM05_) | *CO-linB-F1* | *CO-linB-R1* |
| W1.6 | *Sphingobium japonicum* UT 26 (LinB_UT26_) |  |  |
| W1.7 | *Sphingobium francense* SP+ (LinB_SP+_) |  |  |
| W1.8 | *Sphingomonas* sp SS04-5 (LinB_SS04-5_) |  |  |
| W1.9 | *Sphingomonas* sp SS04-2 (LinB_SS04-2_) |  |  |
| W1.10 | *Sphingomonas* sp. SS04-1(LinB_SS04-1_) |  |  |
|  |  |  | |
| G1.1 | *Sphingobium indicum* B90A (LinB_B90A_) mutant T81A | *CO-linB-F1* | *CO-linB-R1* |
| G1.2 | *Sphingobium indicum* B90A (LinB_B90A_) mutant A83P |  |  |
| G1.3 | *Sphingobium indicum* B90A (LinB_B90A_) mutant V134L |  |  |
| G1.4 | *Sphingobium indicum* B90A (LinB_B90A_) mutant T135L |  |  |
| G1.5 | *Sphingobium indicum* B90A (LinB_B90A_) mutant L138I |  |  |
| G1.6 | *Sphingobium indicum* B90A (LinB_B90A_) mutant H247S |  |  |
| G1.7 | *Sphingomonas* sp. SS04-3 (LinB_SS04-3_) mutant A83P |  |  |
| G1.8 | *Sphingomonas* sp. NM05 (LinB_NM05_) mutant A247H | *CO-linB-F2* | *CO-linB-R1* |
|  |  |  | |
| G2.1 | *Sphingobium indicum* B90A (LinB_B90A_) mutant T81A/A83P | *CO-linB-F1* | *CO-linB-R1* |
| G2.2 | *Sphingobium indicum* B90A (LinB_B90A_) mutant V134L/T135L |  |  |
| G2.3 | *Sphingobium indicum* B90A (LinB_B90A_) mutant V134L/T135L/T81A |  |  |
| G2.4 | *Sphingobium indicum* B90A (LinB_B90A_) mutant L138I/H247S/I253M |  |  |
| G2.5 | *Sphingobium indicum* B90A (LinB_B90A_) mutant A81T/A83P |  |  |

| **Primer Name** | **Sequence (5’ - 3’)** |
| --- | --- |
| *CO-linB-F1* | GGG GAC AAG TTT GTA CAA AAA AGC AGG CTT AAT GAG CCT GGG CGC GAA AC |
| *CO-linB-F2* | GGG GAC AAG TTT GTA CAA AAA AGC AGG CTT AAT GAT TCT GGG CGC GAA CG |
| *CO-linB-R1* | GGG GAC CAC TTT GTA CAA GAA AGC TGG GTA TCA TTA CGC CGG ACG CAG |
